# Diagnostic Challenges in Antiphospholipid Antibody–Associated Chorea: A Case Report

**DOI:** 10.1155/crnm/7560283

**Published:** 2025-10-03

**Authors:** Alexis Robin, Timour Vitte, Lorella Minotti, Philippe Kahane

**Affiliations:** ^1^Neurology Department, University Hospital of Grenoble, Grenoble, France; ^2^CHU Grenoble Alpes, Grenoble Institut Neurosciences, Inserm, U1216, Université Grenoble Alpes, Grenoble 38000, France

**Keywords:** antiphospholipid antibody, fluorodeoxyglucose positron emission tomography, generalized chorea

## Abstract

Choreas associated with antiphospholipid antibody syndrome (APS) are rare, and those linked to transient antibody positivity without APS are even rarer. This case report presents a unique and instructive instance of generalized chorea in an elderly patient associated with antiphospholipid antibodies, highlighting the diagnostic value of FDG-PET in such an uncommon case, which may help guide an etiology-based therapeutic management.

## 1. Introduction

Chorea associated with antiphospholipid antibody syndrome (APS) is a rare clinical manifestation. Even more uncommon are cases of chorea occurring in the context of transient antiphospholipid antibody positivity without fulfilling the diagnostic criteria for APS. This report describes such an atypical presentation, emphasizing the importance of advanced imaging and serological investigations in the etiological evaluation of chorea.

## 2. Case Description

An 80-year-old right-handed woman presented with a 1-month history of generalized chorea with asymmetric choreiform movements of the limbs and oromandibular region without dystonia. She had no personal or family history of neurological conditions, no infection history in the previous weeks, no thrombotic/embolic events, and no pregnancy morbidity in the past. No behavioral or cognitive impairments were identified.

**Figure 1:**
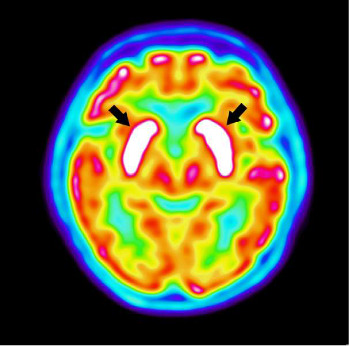
Fluorodeoxyglucose positron emission tomography (FDG-PET) with an intense and diffuse bilateral hypermetabolism in the striatum (black arrows).

Brain MRI including diffusion, FLAIR, T1, and T2 sequences showed no abnormalities to explain her symptoms, with no evidence of stroke in particular. Blood tests, including thyroid-stimulating hormone (TSH), electrolytes, complete blood count, and liver and renal function, were within normal limits. Tests for antinuclear, LGI1, and NMDA antibodies were negative. Anticardiolipin antibodies IgM were positive at 32.1 U in the acute phase while lupus anticoagulant and anti-β2GP1 antibodies were negative. A lumbar puncture revealed no abnormalities in biochemistry, cytology, or bacteriology, with no oligoclonal bands and negative antineuronal antibodies. A thoraco-abdomino-pelvic CT scan showed no lesions. Fluorodeoxyglucose positron emission tomography (FDG-PET) revealed intense and diffuse bilateral hypermetabolism in the striatum (see Figure 1).

The patient was treated with risperidone at 0.5 mg on the first day, increased to 1 mg the next day, for its antidopaminergic effects and due to its simpler use compared to other types of immune-mediated treatments. Improvement, with near-complete resolution of chorea, was observed within 48–72 h. Upon discharge, after 10 days of hospitalization, she no longer exhibited abnormal movements, allowing her to return home. At the 1-month follow-up, the patient was asymptomatic, and we initiated a gradual reduction of risperidone. At the 3-month follow-up, she remained asymptomatic despite the discontinuation of risperidone and anticardiolipin antibodies, both IgM and IgG, were negative. We considered the diagnosis of antiphospholipid antibodies–related chorea.

## 3. Discussion

Chorea is rare in patients with positive antiphospholipid antibodies, with a prevalence of 1.3% in Europe for chorea related to APS [1]. FDG-PET is useful for diagnosing and managing chorea, showing hypermetabolism of the contralateral striatum in hemichorea and bilateral hypermetabolism in generalized chorea associated with antiphospholipid antibodies [2]. Bilateral striatal hypermetabolism is also seen in Sydenham's chorea and chorea due to hyperthyroidism [3]. In contrast, striatal hypometabolism is found in neurodegenerative choreas such as Huntington's disease, neuroacanthocytosis, McLeod syndrome, or spinocerebellar ataxia. Previous reports indicate a regression of striatal bilateral hypermetabolism on follow-up [4].

The pathophysiological mechanisms underlying this FDG-PET hypermetabolism in the context of antiphospholipid antibodies remain debated. Some authors suggest that infiltrating lymphocytes lead to increased glucose uptake [5], while others propose that striatal hypermetabolism reflects a compensatory mechanism that leads to chorea resolution [6]. In this case, the rapid and complete resolution of chorea supports the idea of an ongoing compensatory mechanism at the time of treatment. We also cannot rule out that the transient positivity of antiphospholipid antibodies is the cause of the chorea.

The novelty of our case is that chorea occurs during a transient positivity of antibodies in the absence of definite APS and without alternative diagnosis. In addition, this case highlights the importance of FDG-PET in managing patients with chorea who lack neurodegenerative and behavioral features and have negative initial investigations. FDG-PET may help to guide further biological investigations to identify the underlying etiology, as antiphospholipid antibodies in this case.

In conclusion, FDG-PET is invaluable in the etiological investigation of chorea, whether generalized or hemichorea, significantly narrowing the differential diagnoses in cases of striatal hypermetabolism.

Informed consent was obtained from the patient for this case report. The data have been processed in accordance with the General Data Protection Regulation. No other regulatory procedure in France is applicable to case reports.

## Nomenclature

APS: Antiphospholipid antibody syndrome
FDG-PET: Fluorodeoxyglucose positron emission tomography
TSH: Thyroid-stimulating hormone
